# Qualitative analysis of an intensive care unit family satisfaction survey in Norway

**DOI:** 10.1186/2197-425X-3-S1-A712

**Published:** 2015-10-01

**Authors:** R Lind, B Nerskogen

**Affiliations:** University Hospital of North Norway, Intensive Care Unit, Tromsø, Norway; University of Tromsø, Faculty of Health Sciences, Master in Nursing, Tromsø, Norway

## Introduction

Most patients survive an Intensive Care Unit stay, but due to the illness itself or sedation they might have impaired cognition of the ICU stay. Thus it is difficult to explore these patients' experiences of quality of care and treatment. Their closest relatives, however, often visit the patient and spend time bedside. A study has shown correspondence between patients' and relatives' experiences [1]. Relatives' experiences of ICU care can therefore be important in assessing quality of care. Knowledge about relatives' experiences of care and treatment given to the patient can be used to improve the quality of ICU care [2], [3].

## Objectives

To describe the qualitative findings from a family satisfaction survey, in order to identify the main themes of relatives' ICU experiences.

## Methods

152 relatives of eligible ICU patients in a ten-bed ICU unit completed a Family Satisfaction Survey (FS-ICU 24), which included three open-ended questions on strengths and weaknesses of the ICU unit based on their experiences and perspectives. Responses to these questions were coded and analysed using a comparative method. Data from relatives of ICU survivors and non-survivors were analysed separately and then compared.

## Results

Five domains recur in comments from both relatives of survivors and non-survivors: comprehensive care for them and for the patient, feeling confident by meeting highly skilled nurses and physicians, the need for structured, more frequent and uniform information, the need for better continuity in nurses' and physicians' care, and quality of patient room and the family facilities. The two populations of relatives showed differences in that those about to lose their loved one were more tense and fragile and considerable care was needed in conversations with them. Some distinguished between caring and less caring nurses and physicians, and they also experienced an information gap despite knowing they had been informed.

## Conclusions

The study provided important understanding of why relatives are satisfied or dissatisfied with particular aspects of ICU activities, care and personnel. This knowledge can be used to improve especially the ICU in Tromsø, Norway, but hopefully the results are transferable and useful for other ICUs in enhancing their care.

## FUNDING

University Hospital of North Norway
